# A Hardware-Deployable Neuromorphic Solution for Encoding and Classification of Electronic Nose Data

**DOI:** 10.3390/s19224831

**Published:** 2019-11-06

**Authors:** Anup Vanarse, Adam Osseiran, Alexander Rassau, Peter van der Made

**Affiliations:** 1 School of Engineering, Edith Cowan University, 6027 Perth, Australia; a.osseiran@ecu.edu.au (A.O.); a.rassau@ecu.edu.au (A.R.); 2 Brainchip Inc., Aliso Viejo, CA 92656, USA

**Keywords:** SNN-based classification, neuromorphic olfaction, bio-inspired electronic nose systems

## Abstract

In several application domains, electronic nose systems employing conventional data processing approaches incur substantial power and computational costs and limitations, such as significant latency and poor accuracy for classification. Recent developments in spike-based bio-inspired approaches have delivered solutions for the highly accurate classification of multivariate sensor data with minimized computational and power requirements. Although these methods have addressed issues related to efficient data processing and classification accuracy, other areas, such as reducing the processing latency to support real-time application and deploying spike-based solutions on supported hardware, have yet to be studied in detail. Through this investigation, we proposed a spiking neural network (SNN)-based classifier, implemented in a chip-emulation-based development environment, that can be seamlessly deployed on a neuromorphic system-on-a-chip (NSoC). Under three different scenarios of increasing complexity, the SNN was determined to be able to classify real-valued sensor data with greater than 90% accuracy and with a maximum latency of 3 s on the software-based platform. Highlights of this work included the design and implementation of a novel encoder for artificial olfactory systems, implementation of unsupervised spike-timing-dependent plasticity (STDP) for learning, and a foundational study on early classification capability using the SNN-based classifier.

## 1. Introduction

The biological olfactory pathway is one of the most complex sensing systems in the natural world, mainly because of its ability to identify a wide range of odors by processing high-dimensional, multivariate information. This is achieved through neural signal representation of odor information from millions of receptor neurons and the use of specialized biological neural networks enabling classification of odors in real time [1,2,3]. In order to emulate these processing and sensing capabilities, artificial olfactory systems, also known as electronic noses (e-noses), were introduced in 1982 as chemosensing instruments. Since their introduction [4], e-nose systems have found applications in various fields, including bio-security, environmental monitoring, food quality control, and medical diagnosis [5,6,7,8,9,10]. However, the implementation of traditional processing methods carries with it several limitations, such as poor classification accuracy, high memory and power requirements, and data-intensive complex processing, that have impeded their application as real-time standalone systems [11].

Recent developments in chemical sensing materials along with advances in bio-inspired neuromorphic engineering have shown promising solutions to overcome the limitations of electronic nose systems based on traditional approaches. Neuromorphic olfaction aims to emulate the neuro-computational principles of the biological olfactory pathway in electronic olfactory systems to develop reliable, robust, real-time, and low-power machine olfaction solutions [2,12,13]. The analog very large-scale integration (VLSI) olfaction chip proposed by Koickal et al. in [14] was among the first neuromorphic olfactory systems, comprising of a polymer-based sensor array, a signal conditioning unit for data transformation, and a spiking neural network (SNN)-based classifier implementing spike-time-dependent plasticity (STDP) for learning. Following this research, several other spike-based olfactory systems, such as [1,12,15,16], were proposed, mainly focusing on emulation of the biological olfactory pathway, hence resulting in impractical designs that provide results only under certain operating constraints and, therefore, may not be suitable for real-world applications. Other major contributions in neuromorphic olfaction were reviewed and tabulated in [11].

Recent developments in neuromorphic olfaction have focused on leveraging the sparse spike-based data format to implement practical solutions using novel data-to-spike encoding methods, SNNs as pattern-recognition engines, and bio-inspired learning for classifier training. However, these implementations have not been able to address issues such as computational power requirements, and processing latency [11,13,17]. Through this study, we present an SNN-based solution for electronic olfactory systems that can be implemented on a neuromorphic hardware system for the rapid classification of odors with minimal processing latency. Furthermore, we also introduce AERO (address event representation for olfaction), an address event representation (AER)-based encoder that encodes sensor responses to meaningful spiking data. The SNN was implemented using BrainChip’s Akida Development Environment (ADE) [18], a Python-based emulation of the Akida neuromorphic system-on-chip (NSoC) [19]. The classification performance of the SNN was validated using the benchmark dataset [20] under different scenarios that test the robustness of the network. Furthermore, we also tested the SNN on a partial dataset with reduced timepoints to discuss the potential of implementing the network for early classification results.

## 2. Model Design

Generally, studies focusing on the implementation of neuromorphic approaches for artificial olfactory systems have emphasized emulating parts of, or the entire, biological olfactory pathway to develop data-to-spike encoding and spike-based processing models [11,13]. While such systems can simulate the biological system in detail, they have not been widely adopted for real-world applications due to factors, such as computational and power requirements, and their limited scope for integration into traditional electronic nose systems [11,17]. The SNN-based model proposed through this work introduces the use of event-based encoding and processing of olfaction data to develop a neuromorphic backend for artificial olfactory systems that can deliver reliable classification results with minimal latency and facilitates easy integration into current systems.

The primary aim of this work was to study the existing neuromorphic computing models and implement an SNN-based solution for rapid classification of multivariate olfactory data. We based our model on the SNN architecture provided by the Akida Development Environment (ADE) (BrainChip Inc., Aliso Viejo, USA) [18]. Since the ADE network optimally processes spiking data represented in the address event representation format, one of the key challenges was to encode the olfactory data into event-based spikes that could be further provided as an input to the SNN for learning and classification. This was achieved by developing the AERO (address event representation for olfaction) encoder, a data-to-events encoder that discretized the normalized input signal and generated spikes by encoding the timesteps, activation levels of the signals, and the sensor ids within the array.

The system is based on a three-stage architecture that comprises pre-processing, transformation, and association. Each sensor element within the sensing array of an electronic nose system has distinct responses to different chemical compounds depending on its intrinsic characteristics. Hence, during the pre-processing stage, we implemented a feature extraction approach based on the relative change in resistance for sensor responses with respect to their baseline, a de facto gold-standard feature extraction method implemented in several electronic nose systems [11,21,22,23,24] that provides information related to their characteristic responses when exposed to a certain chemical compound. Following this, the extracted data is normalized in order to rescale the responses so that all values are within the range of 0 and 1.

The subsequent transformation stage, where the input data is converted to event-based spiking data, is a critical component of this model. In this stage, the normalized relative resistance curves are discretized and information related to the activation level of the sensors at each time-point is encoded, along with the sensor ID, into the spiking domain. The discretization of the signal is crucial for the network to implement learning whereby the feature characteristics are partitioned using bins and categorized with the one-hot encoder [25].

**Figure 1:**
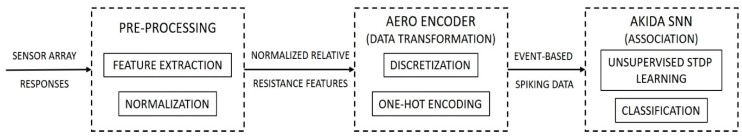
Block diagram of the proposed model using Akida spiking neural network (SNN).

The final association stage of the model is implemented using Akida SNN. The network consists of a single processing layer of fully-connected leaky integrate and fire (LIF) neurons. The network learns via the Akida built-in learning algorithm, an adaptation of the STDP learning approach found in the brain, modified for efficient implementation in low-bit width architectures (see e.g., [26]). Effectively, neurons learn to respond to features that repeatedly present (correlated) over multiple input samples and competition between neurons ensures that they each learn different features. The classification results are determined by implementing a winner-takes-all (WTA) logic, a computational approach that implements a competitive mechanism among the neuron population in the network to determine the classification result based on the activation levels of neurons [27]. Figure 1 provides a conceptual block diagram of the three stages of the system and the computations that take place within the model.

## 3. Materials and Methods

### 3.1. Input Dataset

The input dataset utilized for training and testing of the event-based SNN classification system was extracted from the CSIRO (The Commonwealth Scientific and Industrial Research Organisation) data access portal [20]. This multivariate dataset is recorded using the commercial FOX 3000 e-nose system equipped with a 12-sensor array that consists of sensor elements with a combination of distinct sensitivity and selectivity characteristics. The measurements were performed for 20 different chemical compounds belonging to four chemical groups: alcohols, aldehydes, esters, and ketones, and with concentrations in the range 1.22–8.03 × 10^−5^ M. Sensor responses for 10 samples of each chemical compound were recorded for 300 s at a frequency of 2 Hz, thus resulting in 200 files overall consisting of measurements for 600 time points. Detailed information related to the procedure and laboratory conditions for the e-nose measurements can be found in [24]. This dataset was chosen mainly because it could be used to test the performance of the SNN classifier based on the classification of individual compounds (20-class dataset) and chemical group for each compound (4-class dataset), thereby evaluating the model for different scenarios with different levels of complexity.

### 3.2. Pre-Processing/Feature Extraction

**Figure 2:**
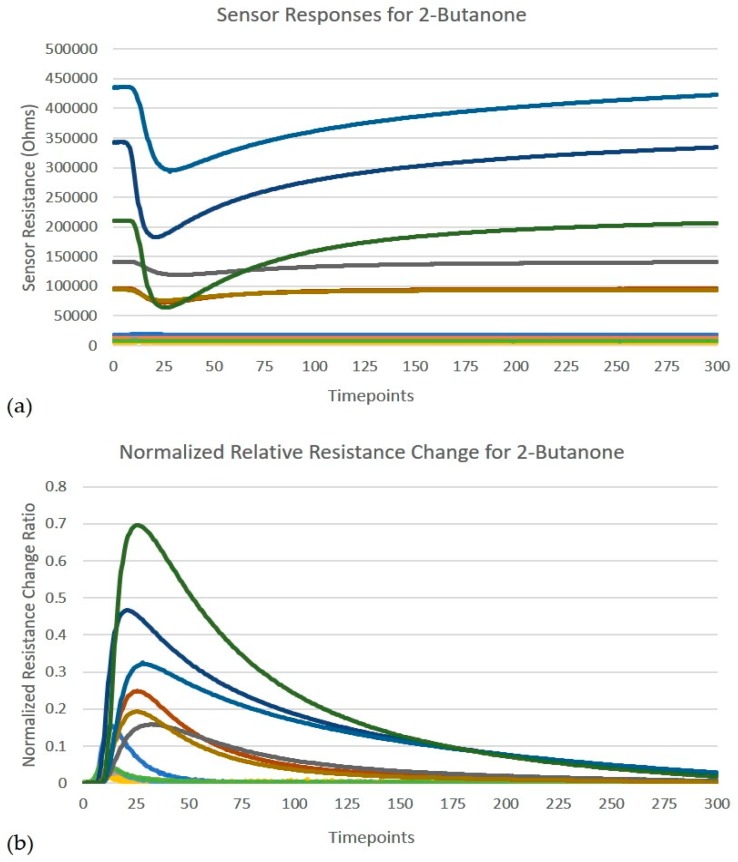
Feature extraction based on relative resistance change. (**a**) Represents the raw timeseries response of the 12-sensor array for 2-butanone (**b**) Shows normalized relative resistance curves after feature extraction for the same sample.

The implementation of classification techniques directly on raw time-series responses from a sensor array results in high-dimensional patterns that are complex to process and result in poor classification accuracy. Furthermore, different sensor elements in an array provide diverse signal types over a significantly large range depending on their sensitivity and selectivity characteristics [23]. As a result, a feature extraction approach was required to obtain discriminative information from the multivariate first-order time responses of the sensor array. The use of steady-state responses to extract the absolute values of relative change in resistance with respect to the baseline is a well-established approach for feature extraction in electronic olfactory systems [22,23]. We applied the following mathematical transformation to the dataset to extract normalized relative resistance change from sensor responses:

(1) $$R_{nm}=\;\frac{R_{i}-\;R_{0}}{R_{max}-\;R_{0}}$$

where, $R_{nm}$ is the normalized response of sensor m, $R_{i}$ is the resistance of that sensor at instance *i*, *R*_0_ is the baseline resistance for the sensor element, and $R_{max}$ is the maximum resistance for the sensor. The normalized relative resistance curve extracted from raw sensor responses for each of the 12 sensors is shown in Figure 2.

### 3.3. AERO Encoder

**Figure 3:**
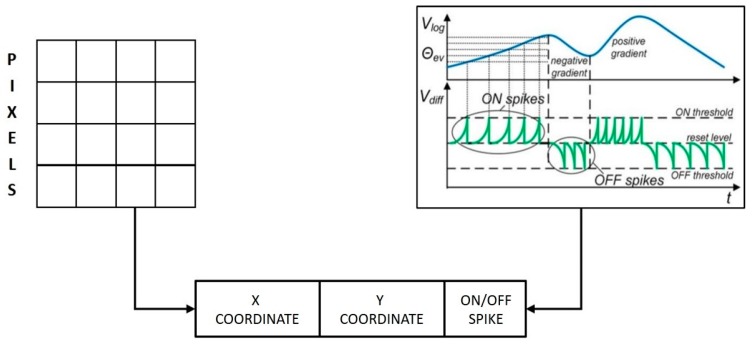
AER spike data format implemented in DVS [28].

The event-based approach for neuromorphic sensing and processing was first conceptualized during the implementation of the dynamic vision sensor (DVS) where events were generated consisting of x-y coordinates of a pixel and ON or OFF spikes based on its luminosity changes [28]. The data structure of the event-based format, also known as address event representation (AER) [29], has been widely used in neuromorphic sensing and processing systems. Moreover, the implementation of an AER bus for neuromorphic hardware ensures maximal transfer rate and spike-time precision of transmitted events. These factors have reinforced the AER format as the de facto standard for neuromorphic applications [17]. Figure 3 shows a schematic of the event format used in DVS.

Generally, bio-inspired electronic nose systems have focused on implementing rate coding or rank-order coding to encode olfaction information into spiking data [11,13]. The idea of an AER-based event-driven approach for neuromorphic olfactory systems has only been applied in studies focused on the neuro-modelling of the biological counterpart [30] and, therefore, its utilization for real-world applications has been limited.

Taking input from previous AER-based implementations in neuromorphic olfaction, we developed the AERO encoder based on the discretization of input signals and the use of the AER data format to encode signal activation levels of each sensor at different timepoints. The operation of the encoder is based on the transformation of normalized relative resistance curves into events using a simple mapping of the values to neuron identities. Each value in the time course of the signals is translated into the AER spiking data format in a 3D matrix, where the x-value corresponds to the timestep, the y-value encodes the amplitude of the signal, and the feature-value is used for the sensor ID. The number of bits used for the discretization of the signal amplitude is crucial to define the specificity and generalization of the activation levels in the subsequent network learning stage based on one-hot encoding approach (example shown in Figure 6.)

**Figure 4:**
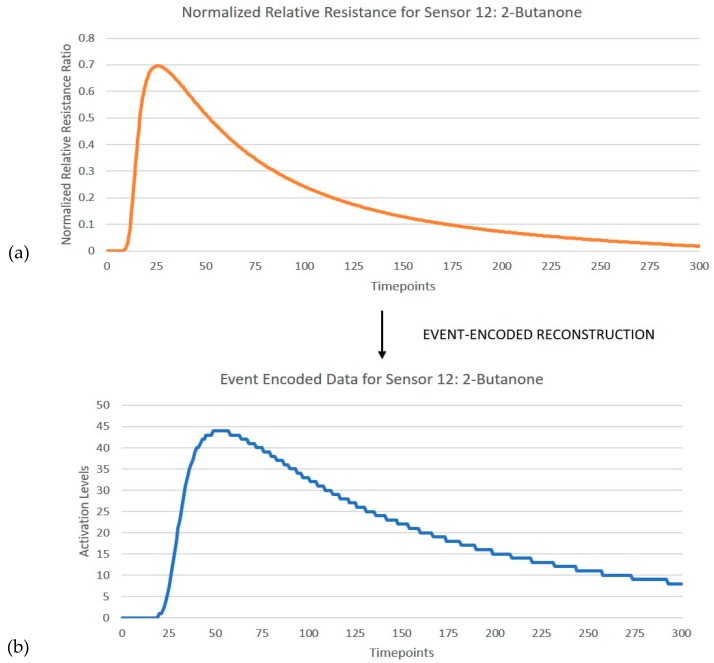
(**a**) Shows normalized relative resistance curve for sensor 12 when exposed to 2-butanone (**b**) Encoding of the feature extracted curve into 6-bit activation levels.

Based on a grid-search optimization, a 6-bit discretization that partitions the amplitude of the signal into 64 activation levels was found to be the best fit, which in this case related to the best overall classification output of the SNN with minimal complexity in terms of the resolution of discretization. The trade-offs of a lower- or higher-bit resolution for signal discretization are further discussed in Section 4.1. The number of timesteps to be included for data-to-event encoding was reduced to the first 150 s, as the remaining time responses did not provide any useful discriminative information. Hence, for each input frame consisting of 12 sensor responses over 300 timesteps, 3600 events were generated by the encoder. Figure 4 shows an instance of the encoder reconstructed relative resistance curve for responses of sensor 12 when exposed to 2-butanone.

### 3.4. Akida Spiking Neural Network

The SNN architecture developed for this study was developed on the Akida Development Environment, a Python-based, machine-learning framework by Brainchip Inc. to define, train, and test SNN models that can be seamlessly transferred to the Akida NSoC [19]. The critical component of this platform is the Akida execution engine that consists of the Akida neuron model that emulates the functionality of biological neurons in a digital format and the unsupervised STDP algorithm. In addition to native event-based computing, the availability of the appropriate features to allow the easy transfer of the model to a low-power neuromorphic hardware implementation were the main reasons for choosing this platform to implement the classifier model.

**Figure 5:**
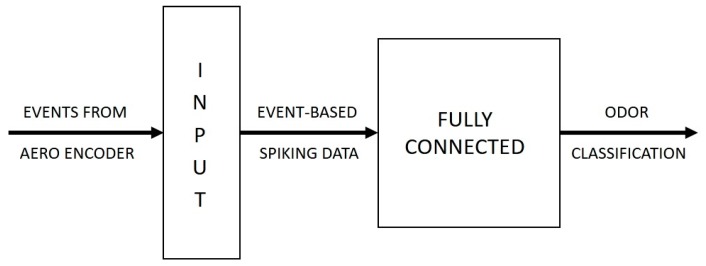
Block diagram representation of the Akida SNN implementation.

The classifier is implemented on an SNN with a simple two-layer feedforward network topology. The first layer is the input layer that receives the event-based spiking input from the AERO encoder and propagates these spikes to the subsequent processing layer. The processing layer, consisting of neurons in a fully connected network, is the layer that is mainly responsible for the learning and classification tasks. Figure 5 illustrates the topology of the Akida SNN implementation.

The Akida execution engine (AEE) requires an input layer to be included as the first layer of the SNN to receive address-event data in the AER data format. Each event is characterized by a trio of values with time-stamp, signal amplitude, and sensor ID values encoded in it. The parameters for the input dimensions, such as the input width, input height, and the number of features, are defined in this layer. The functionality of this layer is to receive event-based inputs within the parameterized constraints and pass them to the rest of the network for processing. Based on the nature of input data, the AEE supports the use of a buffer that can group incoming events and propagate them for processing as packets or directly transfer them for processing as they are received, without any divisions. In this case, the input events are related to a specific test sample and hence are directly propagated through the network upon their reception.

After the input layer, a fully-connected layer is implemented where each neuron is connected to members of the full set of possible inputs. This layer functions as the processing layer and is responsible for learning and classification tasks. A number of parameters are required for the proper functioning of this layer, including the number of neurons to be included for processing and learning, the number of classes that represent the corresponding classes in the dataset, and the number of weights that define the number of connections for each neuron in the network. The learning method implemented for the network has to be defined in this layer, which in this case was set to AEE’s built-in Akida unsupervised learning method. A grid-search optimization was implemented in order to determine the ideal set of parameters that provided stable classification results.

## 4. Results

### 4.1. Classifier Training

**Figure 6:**
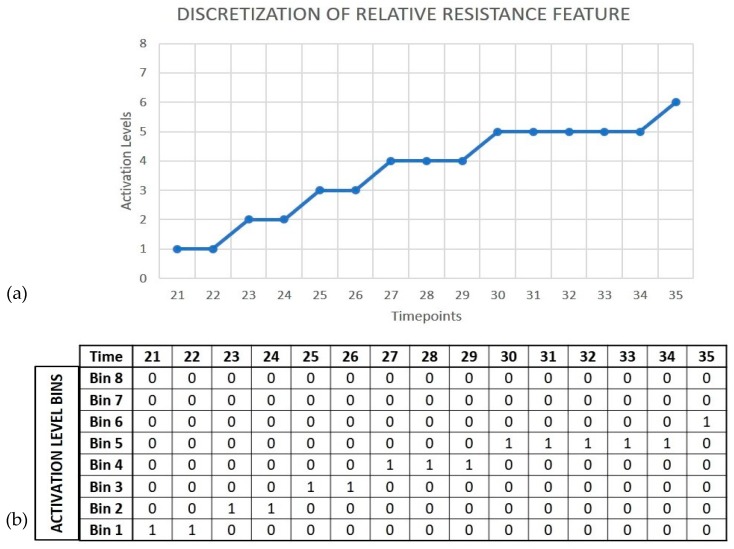
A snapshot of the discretization process. (**a**) The graph shows an 8-level discretization of the normalized relative resistance change features for sensor 11 when exposed to 2-hexenol for 15 timepoints. (**b**) One-hot encoded binning based on partitions of the activation levels.

The discretization of the signal amplitude used for data-to-event encoding plays a crucial role in the network learning process. Based on the number of bits selected for discretization, the signal amplitude is partitioned into 2^n^ levels, where n is the number of bits used. The network learns from features generated by binning the input signals, an approach similar to feature extraction using one-hot encoding, and learns to match the pattern of non-zero bins over time. The level of discretization used controls the binning process, where bins that are fine-grained beyond a certain level result in the network learning very specific patterns, and hence reduces the tolerance of the network to classify samples that show variations from the ones that are used for learning. Alternatively, if the binning is too coarse, the network may generalize samples from different classes and cannot learn sufficient specificity. This approach is illustrated in Figure 6.

The SNN-based classifier was trained and tested for two different scenarios, the first based on the 20 chemical compounds classified individually and the second on the 4-class dataset based on their chemical groups. In each case, 140 samples from the 200-file dataset were used for training and the remaining 60 samples were used for validation. We implemented a balanced 5-fold cross-validation method to ensure that the classifier was tested for each member of the sample set in a specific class.

**Figure 7:**
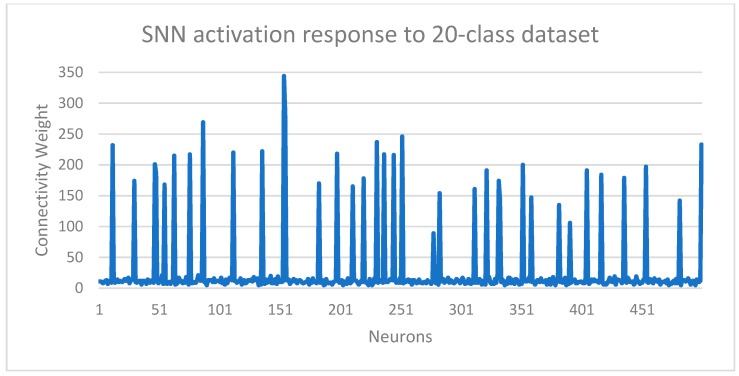
Activation pattern among the neuron population in the SNN. The response shows the resultant changes in connectivity weights for 500 neurons in the network.

The classifier training was activated by enabling AEE’s built-in STDP learning algorithm, while propagating the events generated by the AERO encoder for the training set. STDP is a bio-inspired learning algorithm where the connectivity of neurons in the network is strengthened or weakened based on their spiking activity. As a result, the neurons learn to classify patterns based on their distinct activation responses as well as the overall characteristic response of the network. Figure 7 shows the spiking activity responses of the SNN when events for learning from the 20-class dataset were propagated through the network. Although the Akida SNN is configured for unsupervised learning and learns characteristic features from the correlations in the input data, supervision was added in the form of organization of the neuron population. Each neuron was allocated a class label, which restricted a neuron population to learn from samples of that specific class. Learning was not different from the pure unsupervised case, rather this approach simply avoided the need for a post-learning neuron-labelling step, therefore reducing the training complexity and time cost.

### 4.2. Classification Performance

Once the SNN was trained, the synaptic plasticity changes used for learning were disabled, and the event-based validation data was presented to the network for classification. The SNN classified the input events based on a winner-takes-all logic where the class label of the neuron with the highest activation level among the population was allocated to the presented data. Experiments were conducted for three different scenarios based on increasing complexity. In the first case, the input dataset was classified based on individual chemical compounds, i.e., classifying the dataset into 20-class labels. In the next scenario, experiments were based on a 4-class dataset where the classification of chemical compounds was based on their corresponding chemical groups. Compared to the 20-class dataset, classification of the 4-class dataset increased the complexity of the problem as the SNN and the AERO encoder had to be configured to detect distinctive features in a larger dimension space than the number of samples per class for the 20-class dataset. Lastly, the classifier was tested for early identification efficacy by providing only a part of the early sensor responses and, more importantly, without providing the onset timing of the signal.

**Figure 8:**
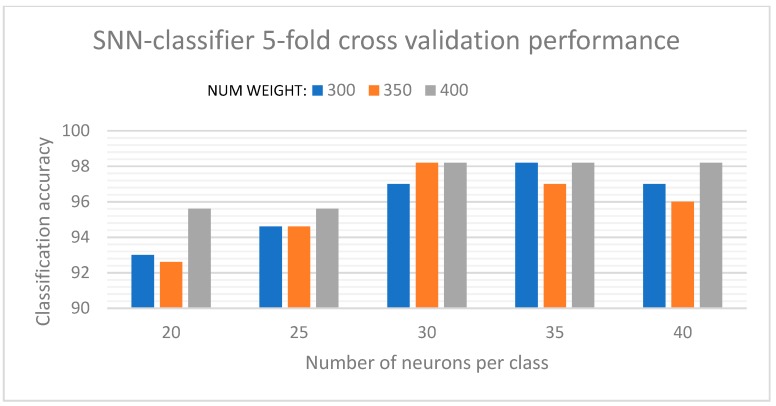
SNN-classifier performance for the 20-class dataset.

Along with the discretization levels used in the AERO encoder, the number of neurons per class, and the weights for connectivity, were the key parameters that influenced the classification results. The optimized parameters were extracted using a grid-search algorithm where the network performance was determined for a specified range of values for each parameter, and the optimum values were selected based on which resulted in the most stable performance of the SNN. The initial plasticity parameter was set to the maximum while initializing the network and gradually decreased based on the neuron activations and learning. For the initial experiments, the SNN provided a stable classification performance of 98.22% for a balanced 5-fold cross-validation. A maximum accuracy of 100% and a minimum accuracy of 96.66% were achieved during these iterations. These results are characterized in Figure 8, which shows instances for the network performance under varying values of number of neurons per class and number of connectivity weights. While the optimum values for the network were determined from a wide range of parameter values, we have included only the key instances of these parameters for which the SNN delivered stable classification results. It was observed that increases in the values of these parameters beyond the optimal range resulted in saturation of the SNN performance and, for some instances, even reduced its performance due to overgeneralization.

**Figure 9:**
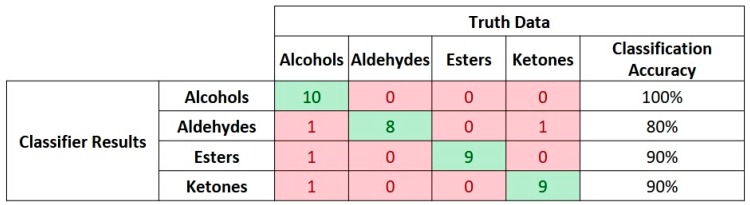
Confusion matrix for SNN-classifier results based on unseen samples for the 4-class dataset.

For the second scenario involving classification of the dataset based on chemical groups of each compound, the discretization levels of the AERO encoder had to be reconfigured and reduced to 5-bit representation in order to allow the SNN to identify discriminative features and generalize the input data. Although the discretization of the input space was reduced, the number of neurons per class had to be increased as the number of events for learning and processing for each class also increased. In this case, the training set comprised 35 samples and the SNN classification performance was validated on the remaining 15 samples from each class. A 10-fold cross-validation accuracy of 96.5% was observed for these experiments with a maximum variance of 2.5% among the iterations. Another set of experiments conducted under this scenario excluded one compound per group during the presentation of learning and testing data. The event-based data from this compound were used to test the SNN classifier performance towards recognizing unseen data without any further modification in the network configurations. In this case, only 4 samples were misclassified among 40 input samples, hence resulting in 90% accuracy. Figure 9 shows the confusion matrix for this classification result.

A classification system that provides primary identification results based on the early response of the sensor elements can be pivotal for electronic nose systems especially considering the relatively slow response of the chemiresistive sensors when compared to their biological counterparts. Moreover, such an implementation is crucial for applications, such as defense and bio-security, where the system may not have access to the entire signal. The final sets of experiments were designed to determine the SNN’s classification accuracy based on early response curves that included the first 30 s of data out of the entire 200 observations in the 20-class dataset. In this case, an instance of the SNN was trained using the time-reduced dataset with 3-bit discretization for the AERO encoder and the network was configured using 20 neurons per class and the number of weights for neuronal connectivity was set to 240. The SNN was trained using 160 samples from the entire dataset and the remaining observations were used as the validation set. The resultant 5-fold cross-validation accuracy using the optimized parameters for the SNN was 92.5%, with a maximum of 95% and a minimum of 90%. These results, based on partial data input, indicate that such an approach combined with the delay-line method discussed in [21], or an increasing window logic [31] can be used to apply the Akida SNN for early classification scenarios.

In general, the key observations for parameters that affected the classification performance of the SNN were: levels of discretization used in the AERO encoder had to be optimal to partition the signal into sufficient numbers of bins based on one-hot encoder logic for learning, a directly proportional relationship was found to exist between the number of discretization levels and the number of neurons per class to efficiently implement learning from the non-zero bins, and weights for connectivity between neurons had to be carefully selected to minimize classification errors due to both overfitting and high-specificity. Table 1 shows the key parameters of the SNN and their values used for the 20-class and the 4-class datasets.

## 5. Conclusions

In this paper, we presented the development of an SNN-based solution for real-time classification of electronic olfactory data. The SNN was implemented using Brainchip’s Akida Development Environment, which provides an emulation of the Akida NSoC’s functionalities on a Python-based software platform. The highlights of this implementation included the development of a novel AER-based encoder for olfaction data, implementation of unsupervised STDP for training the SNN, highly accurate real-time classification results, and preliminary results that lay a foundation for applying the Akida SNN for reliable early classification results.

One of the most significant contributions of this study is the development of AERO, an AER-based data-to-spike encoder for olfaction data. The operating principle of AERO is based on discretizing the sensor responses and encoding their activation levels along with sensor ID and temporal data. For this implementation, normalized relative resistance features were extracted from sensor responses and provided as an input to the AERO encoder. Parameters, such as frequency of event-generation, the number of time points for encoding, and discretization levels, can be configured based on the input data and processing requirements. The development of AERO has opened several avenues for future research, such as encoding multi-dimensional data using different features and interfacing of electronic nose systems with AER-based neuromorphic hardware for processing.

The SNN was tested using the benchmark dataset [20] under four different scenarios of increasing complexity. In general, under each scenario, the classification performance of the SNN was between 90% and 100%, and the processing latency was between 2.5 and 3 s, which includes data-to-spike encoding, learning, classification, and other software-based latencies introduced due to looping and conditional statements. This processing latency would, of course, be dramatically reduced once the classifier is implemented on the Akida NSoC hardware without the overhead of software emulation. Taken together, these classification results show that the SNN-based classifier can deliver highly accurate results with minimal processing latency. Moreover, the ability to transfer the SNN implementation to the Akida NSoC can be leveraged to develop low-power electronic nose systems with minimal computational cost and memory requirements. Intrinsically, in most cases, neuromorphic approaches have proven to outperform traditional processing methods that suffer from limited accuracy, high computational and power requirements, and substantial latency to provide classification results [32]. When evaluated against other neuromorphic and traditional approaches based on the same dataset [21,24,33,34], the results revealed that the SNN classifier developed in this study achieved comparable and, in most cases, better classification performance with minimal computation requirements and latency for both learning and processing. More importantly, the Akida SNN was able to identify patterns from a highly multi-dimensional dataset and classify the dataset based on the four chemical groups of the compounds.

Future research based on these results will focus on the development of a robust SNN-based classifier on the Akida NSoC and its implementation in a real-world application. The efficacy of AERO when combined with rate coding methods, such as [35] and rank-order encoding [2,31,36], will also be investigated. This implementation also lays the foundation for the application of both the AERO encoder and an SNN to study neuromorphic gustation and the fusion of olfaction and gustation for the development of a comprehensive analytical tool for chemical sensing.

## Figures and Tables

**Table 1:** Key parameters and their optimized values for the SNN for the 20-class and 4-class datasets.

Parameter	20-Class Dataset	4-Class Dataset
Minimum Plasticity	0.1	0.1
Learning Competition	0.1	0.1
Number of Neurons Per Class	30	45
Number of Weights	350	410
Plasticity Decay	0.25	0.25
